# An overview of the contribution of fatness and fitness factors, and the role of exercise, in the formation of health status for individuals who are overweight

**DOI:** 10.1186/2251-6581-11-19

**Published:** 2012-10-11

**Authors:** James E Clark

**Affiliations:** 1 Division of Mathematics, Department of Science, Science and Health Careers, MS 29, PO Box 1046, 60 Bidwell Street, Manchester, CT 06045-1046, USA

**Keywords:** Fatness, Fitness, Exercise, Health status

## Abstract

Over the last half century there has been an epidemic of diminished health status induced by what seems as a concurrent rise in a population of individuals that are overfat. During the past few decades, the use of exercise has become a staple in the prevention and treatment options for the retarding the development of health issues pertaining to individuals who are overweight, overfatness or experience obesity. However, there are few studies and reviews look at the global issues surrounding the metabolic and hormone consequences of overfatness and the interaction of exercise with adiposity in humans developing the health status for the individual. This review offers an insight into our current understanding of health issues pertaining to metabolic and hormonal disruption related to overfatness and the treatment effect that exercise, especially resistance exercise, can have on impacting the health status, and overall well-being, for individuals who are overfat, regardless of body compositional changes leading toward a lessening of diseased state, and eventually a return to a normal health status for the individual.

## Background

**Figure 1:**
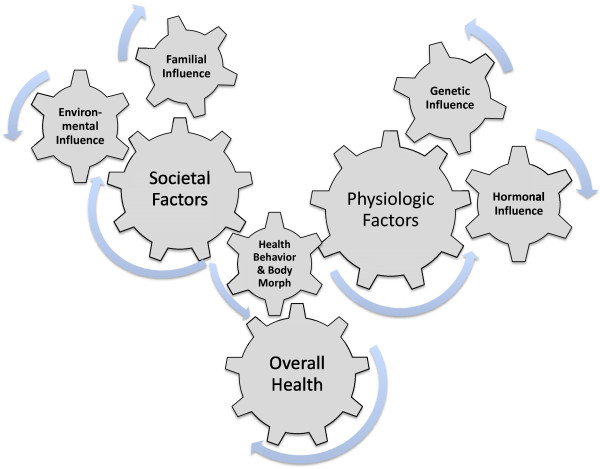
The interrelationship between the various physiological and societal factors, that influence the development of the health behaviors for the individual that in turn impact the overall body morphology and eventual levels of overall health for any individual, regardless of level of adiposity (overfatness) or recognition of obesity.

**Figure 2:**
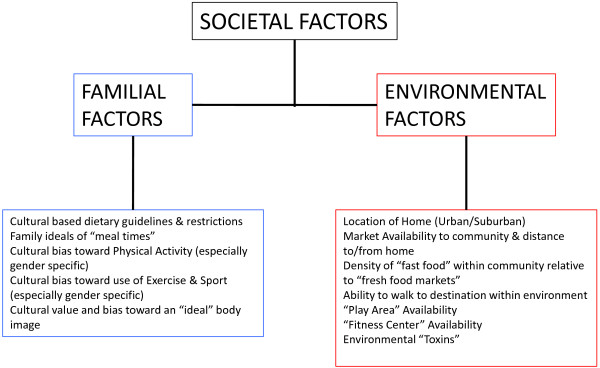
Societal (combination of the familial and environment) factors that contribute the formation of the health behaviors that impact the development of body morphology, e.g., accumulation of fat or fat-free mass, and various factors of fitness which interact to comprise the overall health status for any individual.

**Figure 3:**
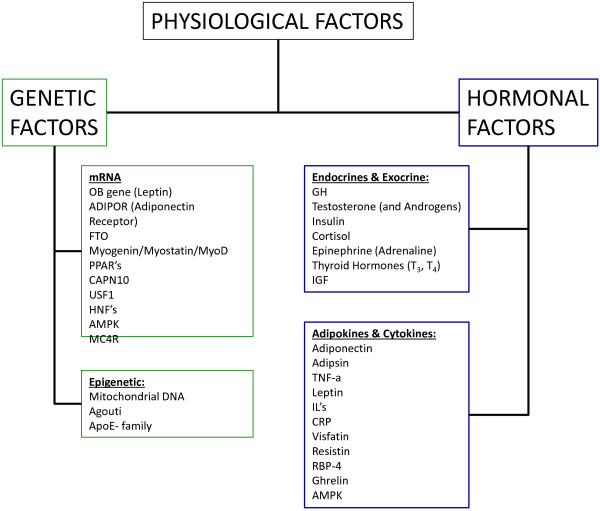
**Physiological** (**Genetic and Hormonal**) **factors that influence on cell physiology and the impact of cell physiology that contribute the formation of the fitness for the individual along with the development of body morphology**, **e**.**g**., **accumulation of fat or fat**-**free mass**, **which interact to comprise the overall health status for any individual.**

Over the past half-century, there has development of an epidemic of health related issues linked with the ever-increasing incidence of overfatness and a concurrent rise of obesity among Americans and populations worldwide [1-3]. During this same time there has been a number of research paths that have identified a number of contributing factors that might be possible explanations for the development this rapid increase proportion of obese individuals within the population, along with the associated health issues attributed to being obese or overfat. These explanations range from the commonly accepted point of view that an excessive intake of dietary calories with a limitation of dietary calories expended through activity over various periods of the individuals life, to changes in genetic and epigenetic regulatory factors, environmental stress and changes in hormonal responses to various physiological stressors, see Figures 1, 2 and 3[1-5]. While it is true that a creation of a positive imbalance in dietary calories will lead to weight gain, this weight gain even if present in the form of fat mass does not by itself develop the “lifestyle diseases”, e.g., cardiovascular disease and metabolic syndrome, which have become the root of the health issues faced by individuals who are overfat.

Instead, the prevalence of health issues seems to be based more on changes in hormone and cytokine signaling (both in production and response) related to the changes in fat mass (even without being visibly obese or overfat) for the individual based on a variability of responses within a continuum of fatness phenotypes, see Figure 2[6-8]. This variability in response necessitates that we be able to differentiate between individuals being “healthy” overweight and “unhealthy” overweight, i.e. overfat or obese, based on the impact of these hormonal changes within their normal physiological responses to stresses of daily living and therefore their overall health status [9]. Additionally, these “lifestyle diseases” that were once solely related to adult and elderly populations, has recently been seen with increasing frequency within the juvenile and adolescent populations, seemingly tied to the increase in the fatness, and a possible concurrent reduction in physical activity and healthy behaviors, of individuals within this population [10-13]. As noted by Fogelholm [13] these change in healthy behaviors within juvenile and adolescent individuals may have long-lasting ramifications, as a reduction in beneficial early health behaviors tend to have a detrimental impact on both health status and activity level throughout one’s lifespan. And can result in changes in various modifications of genetic and epigenetic regulators systemically within the body, particularly at the level of skeletal muscle, liver and adipose tissue, which tend to be involved in the changes to metabolic dysregulation at these same tissues of the body, Figure 1, and can possibility have an impact gamete formation, thus influencing future generations [11,14-16].

This idea of whole population fluctuation in genetic and epigenetic regulation, i.e. the “thrifty gene hypothesis” [15,17] and “thrifty phenotype” [16], has been one of the more popular theories at attempting to explain the current rise in obesity related health issues, especially diabetes. While it may be tempting to lump obesity and overfatness (along with the associated health issues) on genetics, it should be noted at least two reviews on the subject one by Hill and Trowbridge [14] and a more recent paper by Veerman [18] concluded that the rate at which individuals within the population, as a whole, are becoming obese is at an indicated growth rate which could not easily be explained by a distinct genetic cause and that no single gene, or cluster of genes, would have the impact that we are currently seeing in diminished health status within the population of individuals who are overfat, or obese. Whether or not there is a distinct genetic cause for such an increase in the population that is over-fat, there is evidence to indicate distinct hormonal modifications, and thus hormone-genetic interactions, that occurs within the individual based on the increasing adiposity that can lead to the health issues associated with the person being over-fat or obese [5,7,19-23].

**Figure 4:**
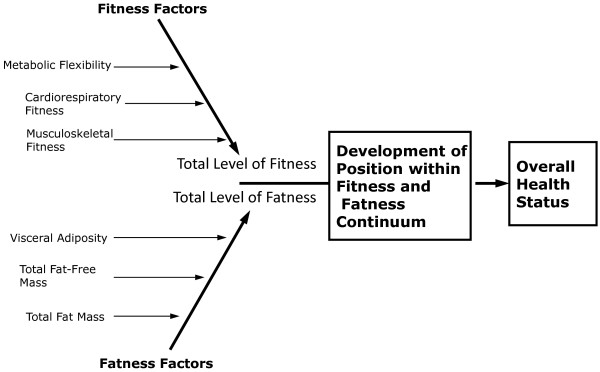
**Interaction between the factors of fitness and fatness the form an inverse relationship combining to eventually determine the overall health status for the individual**, **while the factors and components are identified as individual markers there is actually a highly elaborate web-like interaction between the various factors and is based on the premise of an inverse relationships between Total Fitness and Total Fatness where a high Total Fitness leads to improved overall health status (e. g., low disease state) and High Total Fatness leads to a diminished overall health status (e. g., high disease state).**

**Figure 5:**
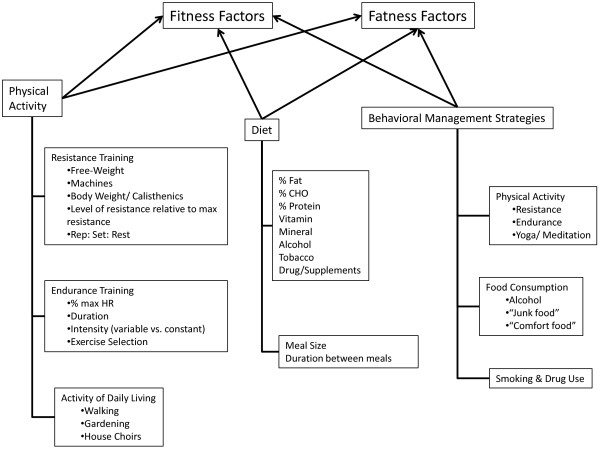
**Outline of the factors that act as components of health behaviors influencing the various factors of fitness and fatness determining the Total Fatness and Total Fitness of the individual and therefore the overall health status for an individual.** LEGENED: % CHO:amount of carbohydrates in diet, % Fat: amount of fat in diet, % Protein: amount of protein in diet.

**Figure 6:**
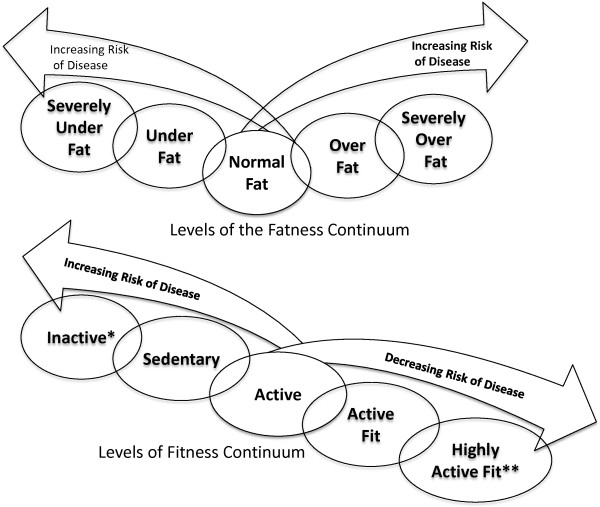
**Examination of the continuum of the levels of fatness and fitness as they relate to an increase or decrease of relative risk for a diseased status for the individual’s health status regardless of observed level of adiposity.** With each increase in level of physical activity, there appears to be an additive improvement to the reduction in the risk for disease state even with expression of high fatness. Note for fitness continuum* the more sedentary a person is they will perform only the minimal activities beyond those of daily living where as Inactive people only perform those activities of daily living. ** the difference between Active and Highly Active is # of day/week of activity.

This interaction between adiposity and hormone-gene interaction appears to cause convergence numerous physiological factors, especially energetic metabolic and immune function, which seems to ultimately determine an individual’s overall health status. This convergence of physiological factors has been labeled by Gruberg [24] and subsequently by McAuley & Blair [25] as the obesity paradox, or by Blair, Cheng, & Holder [26] and other authors [1,27-29] as “fat-but-fit” and “healthy obese”, which will be referred to henceforth as “fit-fat” and seems to be an individualized and related to the interaction fitness and fatness factors, see Figures 1, 2, 3, 4 and 5, along with a continuum of possible responses that formulate the health status for the individual regardless of level of visible adiposity, see Figure 6. In regard to this individualized response, it should be noted, that these fitness and fatness factors combine to establish one’s health status and not necessarily one’s observable level of adiposity, a common and popular misconception [4,10,13,25-33]. Thus indicating that simple visual examination of the person can often mislead both medical professional and research to an incorrect assumption regarding diseased status for an individual based on the observable level of adiposity.

While noting that there is this convergence between the factors of fitness and fatness, we have yet to definitively examine, especially for humans, how the development of health status is impacted by the fluctuations in adipokine and hormone interaction with peripheral tissues that has been linked with overfatness can lead to both the diseased state and eventual resolution of the diseased state, especially via increased physical activity particularly through the use of resistance exercise. While we have definitively shown through numerous animal models that this interaction exists with a relative dose response leading to the disease state and resolution of disease state, we have yet to make the same definitive claims for humans. Therefore the purpose of this review is to examine the response of hormones and adipokines acting at the peripheral tissues are related with the pathophysiology associated with overfatness and the changes seen in these signals with the employment of exercise (particularly resistance exercise) in the resolution of pathologies associated with overfatness leading to improvements in health status for the individual.

### Role of adipose tissue and overfatness in the change of health status

**Figure 7:**
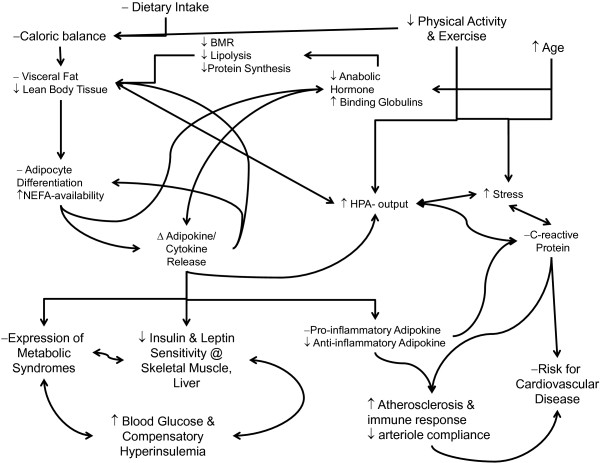
**Interaction between increased caloric balance, reduced physical activity and exercise, increasing age, and/or increased level of psychosomatic stress on the hormonal influences for the development of hyper adiposity (i.e. over fatness and obesity) with reduction in lean body tissue and the subsequent downstream regulatory issues at the various tissues of body (e. g., adipocytes, skeletal muscle and liver) causing changes in production and secretion of deleterious cytokines, interleukins and adipokines that provides the basis for the eventual development of symptoms of metabolic syndrome and cardiovascular disease.** Note that the reduced anabolic hormones (e.g., growth hormone, testosterone, Thyroid hormones, IGF) in production, release and response at the peripheral tissues and increased hypothalamic-pituitary-adrenal output (e.g. increased Epi/Norepi and Cortisol) leads to the downstream issues in series with the increase in visceral fatness from the simple increase in caloric balance (i.e. high dietary intake and low caloric expenditure).

For many years, it was considered that adipose tissue was an inert tissue for the body that was simply involved with the storage of fuel sources (lipids), from periods of feasting, for later use by the body during periods of fasting. Over the past decade, this idea of adipose is an inert tissue simply storing lipids for later use by the body has slowly changed, as researchers have come to a general conclusion that all adipose tissue is not the same and that differentiation of cells within the adipose tissues leads to a change in the behavior of adipose tissue in response to hormones and growth signals associated with many dilatory aspects of one’s health status throughout life, see Figure 7[6,19,20,34-40]. This change in perspective has led to number of researchers asking what appears to be a pertinent question related to the current burden on the health care system based on what is called an epidemic of obesity. What exactly are the hormonal changes and pathophysiological issues related to increased and decreased levels of fatness that leads to diseases, and resolution of diseases, that many individual who are overfat, or obese, face on a daily basis? The initial response to this question began by determining that there is a differentiation of response by the adipose tissue to metabolic stressors and hormonal signaling that results in both hypertophication and hyperplasic changes to the tissue [23,39,41]. It seems to have been established that there are distinct changes in the regulation pulsitility of cytokine (either adipokine or interleukin) releases that have downstream regulation at the various peripheral tissues throughout the body, e.g. skeletal muscle, liver, see Table 1[6,19,22,23,40,42-45]. From this initial response to the question regarding the role of adipose tissue in pathophysiology of “life style disease”, many researchers have begun to speculate about the role of upstream regulatory signals, from adipose tissue, on the downstream metabolic functions within peripheral tissues (e.g., skeletal muscle, liver and bone tissues), and the resulting dilatory changes in health status which tend to result in the accumulation of larger amounts of adipose tissue deposition within the responsive tissues of the body, reduction in cardiovascular function, a state of chronic inflammatory response, and reduced responsiveness to pancreatic hormones (i.e. insulin) and leptin both peripherally at the tissues while also centrally within the cerebral cortex, see Figure 7[8,22,23,30].

**Table 1:** Summary of the key Adipokine/Cytokine associated with health related issues of over-fatness and obesity

**Adipokine/Cytokine**	**Cell/Tissue Secreting**	**Tissue Influenced by Adipokine/Cytokine(Metabolic response to signal)**	**Impact of Over-fatness**	**Impact of Physical Activity**
Adiponectin [46]	A	Skeletal Muscle (↑insulin sensitivity, ↓response to IL-6, TNF-α)	↓	↑
		Liver (↑ insulin sensitivity, ↓response to IL-6, TNF-α)		
		Adipocytes (↑insulin sensitivity, ↓response to IL-6, TNF-α)		
		Cardiovascular Endothelial Cells (↓adhesion formation, ↓response to IL-6, TNF-α)		
Visfatin [6,22,23,43,47]	A	Adipocytes (differentiation of cells)	↑	↓/↔
		Skeletal Muscle (↑ insulin sensitivity)		
		Liver (↑ insulin sensitivity)		
Leptin [6,22,23,40,42,43,47,48]	A	Cardiovascular Endothelial Cells (↑ adhesion formation, ↑ response to IL-6, TNF-α)	↑	↓
		Adipocytes (↑ production and release IL-6, TNF-α)		
		Skeletal Muscle (↑insulin sensitivity, ↑ lipid metabolism, ↓response to IL-6, TNF-α)		
		Hypothalamus/Central Nervous		
		System (satiety, hunger response, indication of energy expenditure)		
Resistin [20,22,49-51]	A, M	Cardiovascular Endothelial Cells (↑ adhesion formation)*	↑	↓/↔
Retinol Binding Protein-4 (RBP-4) [6,43,52,53]	A	Skeletal Muscle (↓insulin sensitivity)	↑	↓
		Liver (↓insulin sensitivity)		
Tumor Necrosis Factor-α (TNF-α) [5,6,45,54-59]	A/M	Cardiovascular Endothelial Cells (↑ adhesion formation)	↑	↓
		Skeletal Muscle (↓insulin sensitivity)		
		Liver (↓insulin sensitivity)		
Interluekin-6 (IL-6) [5,6,20,56,60-69]	M, A/M	Leukocytes (↑ activity and response)	↑	↓
		Cardiovascular Endothelial Cells (↑ adhesion response)		
		Skeletal Muscle (↓insulin sensitivity, ↑inflammation response w/in tissues)		
		Liver (↓insulin sensitivity,↑inflammation response w/in tissues)		
Chemerin [70]	A	Macrophage & Leukocyte (↑ activity and pro-inflammatory cytokine release)	↑	?
		Liver (↓insulin sensitivity,↑inflammation response w/in tissues)		

Note that * indicates that mixed results in outcome of results with research involving human volunteers,^$^ animal studies indicate impact on insulin sensitivity not duplicated with human subjects, A indicates adipose tissue derived chemical, M indicates macrophage derived chemical, A/M indicates both adipose tissue and macrophage derived chemical, ↑ indicates an increase in production and release, ↓indicates an decrease in production and release, ↔ indicates no effect in production and release, and ? indicates unknown response.

While research has linked the altered release of, and response to, adipokines with many of the metabolic changes seen in the body with the accumulation of excessive fat [5,20,43,71], there is unfortunately to date limited evidence to support a conclusive statement regarding the cause and effect relationship of adipokines with the excessive accumulation of body fat, with the exclusion of leptin and adiponectin sensitivity in adipose tissue for humans [8,21,48], or in the reduction anabolic hormones and the relative amounts of lean body tissues that are maintained. However, as shown Figure 7, there appear to be links between the accumulation of fat, with changes in the adipokine and hormone release, state of stress, and a correlative alteration of metabolic processes within the peripheral tissues. The degree of changes are especially noted in the systemic responses to insulin, leptin, and androgen hormones at the peripheral tissues of the body, and the chronic inflammatory state both within the cardiovascular system as well as at the peripheral tissues leading to the changes described in Tables 1 and 2[6,19,20,22,23,37,71].

**Table 2:** Hormones associated with the development and maintenance of lean body tissue and the impact of adiposity and exercise on the production and release by endocrine glands

**Hormone**	**Endocrine Gland**	**Tissue Influenced by Hormone** (**Metabolic response to signal**)	**Impact of Over**-**fatness**	**Impact of Physical Activity** (**Acute**)	**Impact of Physical Activity** (**Chronic**)
Testosterone/Adrenal Androgens [72-82]	Testes Adrenal Cortex	Skeletal Muscle (hypertrophy, add contractile tissue)	↓ with associated ↑ in SHBG	↑ followed by ↓	↔, ↑,↓
		Bone (add bone mass)			
		Adipose Tissue (break-down lipid)			
Growth Hormone [83-86]	Anterior Pituitary Gland	Liver (generate IGF, gluconeogenesis, glycogenolysis)	↓ with associated ↑ in GHBP	↑ followed by ↓	↔, ↑,↓
		Skeletal Muscle (hypertrophy, add non-contractile tissue)			
		Bone (add bone mass)			
		Adipose Tissue (break-down lipid for use as fuel source)			
Insulin [32,44,54,87-96]	Pancreas	Skeletal Muscle (glucose, amino acid, lipid uptake and storage)	↑ (see Figure 8 for rational)	↑ during exercise bout, ↓following	↓
		Liver (glucose uptake and storage)			
		Adipose Tissue (glucose and lipid uptake and storage)			
Insulin-like Growth Factor (IGF) [86,97-103]	Liver, Skeletal Muscle, Bone	Skeletal Muscle (hypertrophy, add non-contractile and contractile protein)	↓ with associated ↑ in IGF-BP	↑followed by ↓	↔, ↑,↓
		Bone (add bone mass)			
Cortisol [60,84]	Adrenal Cortex	Skeletal Muscle (break-down of protein tissue, lipid break-down, decreased use of glucose)	↑(see Figure 8 for rational)	↑ during exercise bout, ↓following	↔,↓
		Bone (break-down of protein matrix)			
		Liver (glycogenolysis)			
		Adipose Tissue (break-down lipid for use as fuel source)			
		Immune Cells (regulation of inflammatory response)			
Triiodothyronine (T_3_) [104,105]	Thyroid	Skeletal Muscle (regulation of metabolism and fuel source utilization)	↓ (Hypothyroidism associated with obesity unknown causal relationship)	↑ during exercise bout, ↓following 60 minutes of recovery	↔,↑
		Bone (regulation of generation of protein matrix)			
		Adipose Tissue (regulation of lipid deposition and utilization)			
Thyroxin (T_4_) [104,105]	Thyroid	Skeletal Muscle (regulation of metabolism and fuel source utilization)	↓ (Hypothyroidism associated with obesity unknown causal relationship)	↑ following exercise bout, ↓following 120 minutes of recovery	↔,↑
		Bone (regulation of generation of protein matrix)			
		Adipose Tissue (regulation of lipid deposition and utilization)			

Note that ↑ indicates an increase in production and release, ↓indicates a decrease in production and release, ↔ indicates no effect in production and release, and ? Indicates unknown response.

There are additional associated pathologies within the cardiovascular system noted with overfatness, e.g., elevated levels of lipids and lipoproteins (dyslipidemia), due to the increased availability of fatty acids from both the diet and release of lipids from adipose tissue throughout the body. This change in lipid availability seems to have little impact on the metabolic use of fatty acids as a fuel source within the body of individuals who are obese, without a concurrent rise in adiponectin levels that is seen only with increasing levels of physical activity [33,35,46,47]. This reduction in metabolic flexibility (noted by a rise in fatty-acid availability without concurrent utilization within the metabolically active tissues of the body) is commonly associated with raised levels in the measures of blood lipids (e.g., total cholesterol measures of HDL, LDL, and vLDL, and triglyceride) levels throughout the cardiovascular system [21,35,46,47]. Additionally, metabolic inflexibility, dyslipidemia along with the associated increase in atherosclerosis plaques and hypertension, appear to be linked with changes in release of adipokines (e.g., adiponectin, leptin, resistin) and cytokines (e.g., TNF- α, IL-6) that are linked to the increased development of endothelial adhesion and reduction of arteriole compliance, see Table 1 and Figure 7, leading to further cardiovascular health issues for the individual throughout their lifespan [6,12,20,71].

Lastly, there is also growing evidence that for individuals with higher levels of adiposity there is a reduction in the production of sex (androgen) hormones and a concurrent rise in the carrier proteins for the various sex hormones (SHBG) [34,38,72,106]. This association between increase in SHBG and reduced free androgen hormones seems to be linked with both the accumulation of fat mass, and reduction of fat-free mass, and has led to a speculation of a link between the changes in available androgens and the associated epidemic rise of metabolic diseases within both juvenile and adolescent populations, as well as adult and elderly population, that express overfatness or are obese, for purported impact see Table 2 and Figure 7[3,34,38,72,106].

All of which leads many to speculate as to a possible role of adipose tissue and circulating adipokines dysregulation and the inability to respond to circulating anabolic and steroid hormones (e.g., insulin, adrenal androgens, growth hormone, estrogens, glucocorticoids, progesterone, testosterone) throughout the time that an individual expresses overfatness.

### Role of fitness, exercise and physical activity behaviors in the change of health status

Fitness, just like fatness, develops through a combination of many facets of one’s lifestyle (behavior) related to the innate qualities of the person such as their genetic and hormonal components establishing metabolic responses to various stresses throughout one’s lifespan, see Figures 1, 4, 5 and 6[6,19,20,107]. The idea of fitness has been evolving over the past few decades as various lines of research has investigated how each component of physiological function leads into one’s overall fitness. Any discussion of fitness must begin with attempting to define a very complex, and ever evolving, issue as to what is exactly meant by fitness. Fitness, defined here, is a multifaceted dimension of tissue and organism function that is composed of the interactions of the cardiovascular, respiratory, immune, nervous, endocrine, and musculoskeletal systems that lead to one’s ability to complete and recover from all tasks of daily living and recreational activities with a minimal level of chronic immune response and inflammatory biomarkers.

As it relates to the individuals who are overfat or obese, any improvement of fitness come from the change in multiple metabolic processes that are linked with release of, and response to, various adipokines, cytokines, and hormones at the peripheral and central tissues of the body, see Tables 1 and 2. While more fatness generally leads to a greater release in deleterious adipokines and cytokines from adipose tissue, improving fitness through increased physical activity (especially resistance exercise) leads to the greater responsiveness to anabolic hormones (e.g., insulin, androgens, growth hormone, IGF, and thyroid hormones) and advantageous adipokines and cytokines (e.g., leptin, adiponectin, and IL-10), along with a concurrent reduction in the release of the deleterious adipokines and cytokines (e.g., resistin, adiposin, TNF- α, IL-6 and RBP-4) and hormones (e.g., cortisol) [7,8,21,48,49,71,83]. These changes in responses to the circulating milieu of hormones, cytokines and adipokines following physical activity tend regulate the metabolic activity of tissues (e.g., skeletal muscle, bone, liver and adipose tissue) which leads to quantifiable improvements in overall health status. This improvement is seen most dramatically in the return to metabolic flexibility, reduction in the chronic state of inflammation found within both the peripheral tissues (e.g., skeletal muscle and adipose) and the cardiorespiratory system for the individual that can lead to a concurrent the increase in lean tissue and reduction in fat mass if chronically employed [4,19,37,43,83,87,88,108-110].

**Figure 8:**
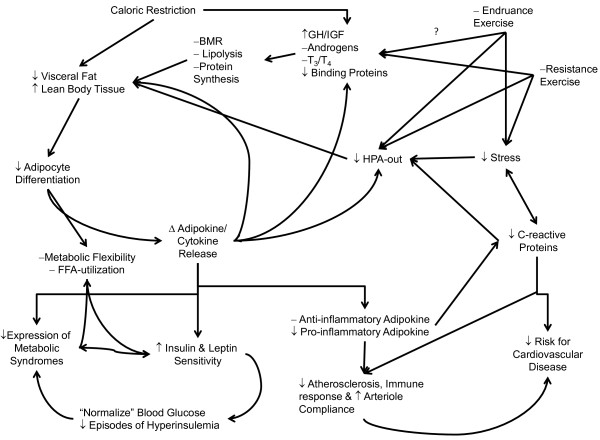
**Interaction between caloric restriction, increased physical activity and exercise, and a reduction in the level of psychosomatic stress on the hormonal influences for the reduction of hyperadiposity (i.e. overfatness and obesity) and increase in lean body tissue, leading to the improvement in subsequent downstream regulatory issues at the various tissues of body (e.g., adipocytes, skeletal muscle and liver) causing changes in production and secretion of deleterious cytokines**, **interleukins and adipokines that provides the basis for the eventual resolution of symptoms of metabolic syndrome and cardiovascular disease.** Note that the improved response in anabolic hormones (e.g., growth hormone, testosterone, Thyroid hormones, IGF) in production, release and response at the peripheral tissues and decreased response at the hypothalamic-pituitary-adrenal axis (HPA) output (e.g. increased Epi/Norepi and Cortisol) leads to the resolution of previous downstream issues in series with the decrease in visceral fatness from the simple caloric restriction leading the resolution of overfatness, metabolic syndromes and cardiovascular disease. Note the ? on the Endurance Exercise pathway as mixed evidence indicates that increasing anabolic hormone release with Resistance Exercise is known while it is still in doubt as to the extent of changes following Endurance Exercise in regards to the release of anabolic hormones.

From these responses, there appears to be a continuum of response with improved fitness being an acute response to the bout of exercise, see Figures 4 and 5, and the desired morphological changes from increased physical activity appear as a chronic response that is the continuation of initial acute changes for the individual leading to improved metabolic function and hormonal responses to a number of physiological stressors, see Figure 8[83,88,107,110-113]. Because of these continuums of events, which can appear after just a single bout of exercise we must ask, how does improvement in fitness affect one’s health status if there is a large degree of fatness? Given all of the health issues surrounding the accumulation of fat mass, just how exactly does improving one’s fitness level counter-act these detrimental health issues? While the principles of exercise as it relates to a change in healthy individuals have been well documented, a question remains as to how exactly does increased fitness counteract the accumulation of fat mass that takes months and years to develop into the overfatness that negatively affects one’s health status?

There is a growing body of evidence to support that most of the body compositional changes that we associate with the change in fitness status comes from the chronic changes to adiponectin, as much as the changes noted to the anabolic hormones, which both have positive fluctuations with increased physical activity for those individual who are overfat or obese [8,54,114]. While others [28,32,54,60,87-89,115-117] have indicated that the primary metabolic adaptations (especially the reduction in insulin resistance) leads to increased metabolic flexibility for skeletal muscle with improvements in insulin and leptin sensitivity throughout the body, and normalization of hormone concentrations. In this regard it is the associated with this change in leptin metabolism is a reduction of both pro-inflammatory adipokines and cytokines (e.g., resistin, leptin, RBP-4, CRP and TNF- α) along with reduction resting levels of cortisol that appears to lead to the resolution of deleterious health issues for the individual who is overfat or obese [6,12,20,50,71,118].

### Treatments and treatment effects

It is this idea of changes in hormonal signaling that has lead to the idea of a treatment effect for exercise at reducing the impact of fatness and increasing the impact of fitness on the development of the individuals health status. Unfortunately there are a number of factors that have hindered our approach to determine the true treatment effect of exercise for those individuals who are overfat or obese. This hindrance is noted in two distinct areas of research, first is our overall ability to quantify and qualify a change in fitness status within the overfat populations stems from the misconceptions of how the general medical population (and some researchers) measures and discusses issues of fitness and fatness and secondly an inability among researchers to appreciate the overall complexity of how exercise (especially the difference in response to endurance and resistance training) truly functions in many of our research models of treatment for the pathology of overfatness and obesity [113,119].

Given the deleterious effects of health that accumulating high amounts of adipose tissues leads to, there has been a concerted effort by the medical and public health communities to counteract the rise of individuals who are overfat or obese within the general population not only in the US but worldwide [3]. Through this effort, there has developed the assumptive position for treatment options that revolve around the simple development of a negative dietary caloric balance that is encouraged to be developed either through dietary calorie restriction within the diet, or an increased dietary calorie expenditure through physical activity, or a combination of the two [29,30,120-124].

While many physicians and health professionals support the use of dietary caloric restriction to reduce body mass, any reduction in fat mass may not be linked with any increase in fitness for the individual. This due to the fact that a development of a negative dietary caloric imbalance through dietary modifications is only one component in a very complex interaction of factors that influence the health status for any individual, especially for those who find themselves as being over-fat, see Figures 7 and 8, as weight gain or loss is a systemic process and never limited to a single tissue (e.g., adipose tissue). In opposition to simple dietary changes leading to weight reduction and a normalization of health issues, researchers have shown that physical activity (as examined primarily through endurance training methods) of at least moderate intensity (50-70% of individual’s VO_2max_ associated HR) leads to both positive adaptations with the cardiovascular system (e.g., reduction of vascular inflammation, cholesterol, glucose and triglycerides levels), an increase in fatty-acid oxidation, and hormonal levels (e.g., improved insulin and leptin sensitivity, reduced cortisol and inflammatory signals) independent of, and prior to, anybody compositional changes that might result from chronic training [8,30-32,44,54,87,108,114,122,125-127]. As described in Figure 8, this differential response may be linked with the additive response of hormonal adaptations that are seen with exercise and physical activity that are not normally seen with simple dietary caloric restriction. Which is supported by a number of researchers, indicating that when caloric restriction is combined with exercise there is a differential reduction of body mass versus the changes seen simple caloric restriction, and when resistance exercise is incorporated into the treatment a retardation of the total loss of lean body mass [30,109,111,120-124,128-130]. In further support of this position, when resistance exercise is incorporated into the behavioral treatment programs, not only is the reduction in mass primarily fat mass, but there resounding effect of the use of exercise on the overall improvement in measures of fitness, e.g., resting blood pressure and cholesterol levels, and abnormal blood sugar levels or metabolic disease for the individual [28,32,54,90,108,117,118,121,131].

Unfortunately for the individuals who are overfat or obese, regardless of how effective any exercise program might be, the self-efficacy of follow through to any program seems to be quite low. This phenomenon has been noted, in private conversations with a number of physicians, and by Eriksson [132] in his review of exercise treatments of individuals with Type 2 Diabetes Mellitus, that up to 90% of self-started exercise programs by overweight individuals are terminated within 12-months of initiation of said program. While there are number of factors that are involved in the aspect of exercise attrition, one key factor appears to the innate attraction to a mode of exercise for the individual and psychological rewards obtained from exercising. Based on the psychological components of exercise selections, rate beneficial adaptations, and general attraction towards distinct patterns of exercise, there seems to be support for the speculation that the individuals who are overfat may have an innate willingness to self-select toward resistance exercise [30,88,129,132,133]. Based on the speculative propensity for, and possible innate drive toward selecting resistance exercise, resistance training may by the simple attrition of all factors provide the greatest benefit for the overfat or obese population. This benefit should be especially true if the exercise regimen incorporates principles of a periodized resistance exercise program that is readily used in exercise prescription for the healthy fit and athletic populations [134,135], and even more so when resistance exercise is utilized concurrently with the moderate-to-moderately-high intense endurance training [49,90,109,115,121,131,136,137].

## Conclusion

Because of the interactions between a variety of factors that contribute to the development and maintenance of body composition it is overly simplistic to continue to use the dietary notion of dietary caloric restriction to reduce body mass as a means to alleviate the epidemic diminishment in health status associated with overfatness and obesity. However, it is the combination of factors, both fitness and fatness, within overlapping continuums that formulates one’s overall health status and that there appears to be a greater benefit for the individual from increasing the level of physical activity rather than the absolute level of fitness. While improving fitness and reducing fatness may lead to an improvement in health status that may or may not make the person as “healthy” as a fit active person; it is speculated here, if physical activity is continued at a high to moderate intensity (especially with a periodized resistance training program) may eventually serve as a treatment medium for total resolution of health-related issues that have arisen from the period of life with overfatness.

However, in order to better understand the complexity of the issue, future research needs to look at the involvement of anabolic and steroid hormone dysregulation within the scope of movement towards and away from disease status as it relates to the accumulation of adiposity. This dysregulation may involve the disruption of pathways that should allow for the deposition of lean body tissue and normal energetic metabolism but may also become involved with the alteration of the genetic machinery of the adipose tissue leading to higher cell turnover, limit apoptosis and a greater differentiation of adipose cells exacerbating the health issues for someone who is over-fat or obese. One of the biggest culprit in this cascade toward a disease state may be the reduction in free anabolic hormones and response at the tissues through regulation of receptors that while seen in the aging population may in fact be at play with the young but over-fat population that can lead to the deleterious health issues that are counteracted by increased physical activity and regimented exercise programs. Lastly, research needs to compare the relative benefits that are attainable from use of resistance versus endurance training, to date we were only able to find three such studies utilizing human subjects. Additionally, we need to complete examinations, not only look at the physiological benefits and improvements in health status but also at the psychology of exercise selection and adherence of the exercise program of choice (or prescription) for individuals who are over-fat or obese.

## Competing interests

The author acknowledges that there is no competing interest in the production of this manuscript.

## Authors’ contribution

JC is the sole author of this manuscript completing all reviews, writing, editing, and synthesis of tables and images contained here.
